# Graduate Study on the Continent—Germany

**Published:** 1908-10-03

**Authors:** 


### HOSPITAL ADMINISTRATION.

### CONSTRUCTION AND ECONOMICS.

### GRADUATE STUDY ON THE CONTINENT—GERMANY.

#### XI.—DENTAL GRADUATE STUDY IN GERMANY.

Amidst the enormous development of medical graduate study, due largely to the strenuous exertions of the Central Committee of whose endeavours we have on more than one occasion made mention, the dentist has not been forgotten. It has been recognised, justly, that in the same way that the average general practitioner needs help to keep himself abreast with modern advances in scientific medicine, so does the qualified dentist feel the need for occasional assistance. Dentistry, like medicine and surgery, is daily progressing; methods of yesterday are being replaced by newer and better ones, and the dentist, to be up-to-date, has to read and learn daily to practise and try various novelties. The full study of theoretic dentistry and its development is as much an impossibility for the practising dentist, as the full study of the latest advances in medicine is an impossibility for his medical colleague. For this reason graduate study in dentistry is not a fad. It is a serious practical matter that demands attention as much as graduate study in medicine demands consideration, and it is interesting to see how Germany has dealt with the matter.

The Central Committee, very early in its career, grappled with this question, and tried to arrange a series of courses for dentists. A sub-committee was formed, at the head of which is Professor Dr. Hahl, and the general secretary of which is Dr. K. Cohn (address, Schriftführer Komitee für Zahnärztliche Fortbildungs Kurse, in Berlin; Kaiserin Friedrich Haus, Luisen Platz, Berlin). This committee regulates all the graduate courses in dentistry in Germany, gives particulars and full information regarding dental study to intending participants and foreign graduates, arranges for lectures and demonstrations and acts as a central station for the rapidly developing dental graduate study movement generally. Dentists who wish to become acquainted with Continental methods cannot do better than apply directly to the secretary for information regarding courses, etc.

In Berlin regular courses in dentistry are given under the auspices of this committee, and a glance at one of the programmes of such vacation courses is not without interest, as it shows the catholic view which the committee has taken. The following, for instance, are a few of the Berlin courses designed for dental post-graduate students:

Professor Albu: Auscultation and percussion in relation to local and general anæsthesia in dental operations; Dr. Kron: nervous diseases and dentistry; Dr. Richter: abnormal conditions of the teeth and jaws and their treatment; Dentist Höxbroe: gold fillings with Abbeys medium; Dr. Dieck: porcelain fillings and prothesis according to Dall's method; Dr. Cohn: local and general anæsthesia in dental practice; Dentist Mex: novelties in technique of dentistry; Dr. Immelman: *x*-rays in dental practice; Dr. Müller: adrenalin, the dangers of local anæsthesia and its addenda; Dentist Mamlok: continuous gum work; Dentist Heydenhaus: abnormalities of the incisor teeth; Dentist Schramm: new methods in porcelain work; Professor Waldeyer: Albert Kölliker and Walter Flemming as odontologists; Professor Warne Kros: causes of dental caries; Professor Miller: caries of the teeth and wedge shaped erosion.

A few items from the courses given at other centres may also be included here.

*Frankfurt.*—Dr. Albrecht: New methods in gold fillings. Dr. Kunert: Redressement forcé; root extraction in difficult cases. Dr. Ollendorff: The Ollendorff method and its application. Dr. Hubner: Diseases of the oral mucosa and their relation to caries of the teeth. Dr. Herxheimer: Dermatology and dentistry. Dr. Dill: New methods in dental technique.

*Kiel.*—Dr. Sye: The Herbst method and its application. Dr. Hentze: Harvadid and Astral, new silicate fillings, etc. Dr. Fricke: New electric apparatus in dental practice. Dr. Hinrichson: Davis crowns and the Jüterbock apparatus; new methods of dental prothesis.

*Strassburg.*—Dr. Römer: Narcosis in dental practice. Dr. Stolky: Surgical operations on the jaws and the dentist. Dr. Hey: Nervous diseases and the teeth; complications of dental neuralgia.

*Greifswald.*—Dr. Bahls: Bacteriology and protozoology in relation to dentistry. Professor Schröder: Aspiration technique in dentistry. Dr. Kollin: Double fillings; complications of multiple extractions. Dr. Behrend: Prothesis immediate and late in cases of resection. Dr. Sagebiel: Diseases of the antrum of Highmore.

These few items, culled from a large and varied programme, show clearly how wide and how thorough the conception of the committee is with regard to dental graduate study. Nor have their efforts been without success. To-day these courses are everywhere crowded, and not only local practitioners, but dentists from all parts of Europe attend them.

As we have no practical experience which warrants our drawing a comparison between German and English methods in dentistry, we shall not endeavour here to point out in what respects the English dentist may learn from his German colleague, and only content ourselves with pointing out what facilities, apart from the above-mentioned courses, exist in Germany for graduate study in dentistry. These facilities are by no means few. The dental student is recognised as a University man; his professors are University professors, and with a few exceptions every University possesses its own "University Dental Clinic." The diplomas which confer the right to practise dentistry are granted by the Universities, after examination, the length of study being four years. Many dentists, however, take a medical degree as well, the degree of doctor in dentistry not being conferred by all the Universities. At Berlin the University dental clinic is an up to date institution which can hold its own with any English rival, while the clinics at Breslau, Leipsic, Munich, Greifswald, Göttingen, and last, but by no means least, Strassburg, are equally good. At these clinics the foreign dentist will find that he is a welcome guest. He will never have to complain of any want of courtesy on the part of his teachers, and he will have a large amount of material to work with, and will be able to acquaint himself thoroughly and in a relatively short time with such methods as may be novel to him.

That there are such methods we do not for a moment doubt. Speaking merely from a surgical point of view, the English visitor is impressed, for instance, with the excellent work which is done in immediate prothesis in cases of resection or partial resection of the upper or lower jaw. In England such cases are operated upon by the surgeon and sent out, to return in a few months' time to the dental department "to see what the dentist can do for them." Very often he is able to do but little, because he has not seen the case before. In Germany a "jaw case" is seen by the dental professor in consultation with the surgeon. Impressions are taken long before the operation, a suitable prothesis is fashioned, and this is inserted at the time of the operation, giving, in the majority of cases, splendid results so far as appearance and what is generally known as "cosmetic effect" are concerned. Where immediate prothesis is impossible or where the surgeon is called upon to resect more widely than he originally intended, the dentist sees the case frequently after the operation, and takes it in hand as soon as possible. This hand-in-hand collaboration with the surgeon is one of the things that strikes the medical visitor most in German dentistry. Another feature is the importance that is attached to an accurate knowledge of anæsthetics—especially local anæsthetics—in dental practice. General anæsthesia has been almost abolished so far as the dentist is concerned, and the ordinary extractions are performed almost entirely under local analgesia. Yet another feature is the extensive use of the *x*-rays and the parabolic lamp in examining the teeth and mouth. The up-to-date German dentist has his electric apparatus in perfect order, and in many cases the consulting-room contains a number of delicate and admirable instruments for diagnostic purposes with which dentists in England are certainly not generally acquainted. Of the immediate technique, methods of filling, extraction, and crowning, we do not presume to speak, but German medicine and German surgery hold so much that is interesting, novel, and richly instructive to the English graduate that it is easily imaginable that German dentistry may equally hold much that would appeal to the English dentist.

For the graduate who is interested in the theoretical side of his work, Germany offers, undoubtedly, many attractions. The pathology of those obscure lesions of the jaws, the cystic and epithelial odontomes or adamantinomes, (as the newer German classification styles them), the interesting treatment of fractures of the jaw, the wide subject of antral lesions and caries—all these points he will find have keenly interested German pathologists during the last half-dozen years, and the work that has been done on these subjects is to a great extent unrecognised with us as yet. He will meet with rarities in the dental clinics, ranging from the loose teeth in cases of permanganate poisoning to extreme ankylosis of the jaw, which tests the ingenuity of the most expert surgeon and dentist combined, and will have a rich field in which to glean observations for future use. What we have detailed in the foregoing chapters regarding living, general expenses and attendances serves for the dental student as well as for his medical colleague. For him also it would be advisable to master the language thoroughly, so as to speak and understand it well, before he attaches himself to a German clinic. There lies no great difficulty, as we have before observed, in obtaining a working acquaintance with German, and the dental student will find a knowledge of the language of practical use afterwards.

